# Comprehensive Identification and Characterization of Long Non-coding RNAs Associated With Rice Black-Streaked Dwarf Virus Infection in *Laodelphax striatellus* (Fallén) Midgut

**DOI:** 10.3389/fphys.2020.01011

**Published:** 2020-08-12

**Authors:** Jian-Hua Zhang, Yan Dong, Wei Wu, Dian-Shan Yi, Man Wang, Hai-Tao Wang, Qiu-Fang Xu

**Affiliations:** ^1^Institute of Plant Protection, Jiangsu Academy of Agricultural Sciences, Nanjing, China; ^2^Key Laboratory of Food Quality and Safety of Jiangsu Province - State Key Laboratory Breeding Base, Nanjing, China; ^3^Nanjing Plant Protection and Quarantine Station, Nanjing, China

**Keywords:** *Laodelphax striatellus*, midgut, Rice black-streaked dwarf virus, long non-coding RNA, RNA-Seq, RNA interference

## Abstract

Long non-coding RNAs (lncRNAs) are involved in a variety of biological functions through transcriptional and post-transcriptional regulation. However, little is known about their functions in the process of insect mediated virus transmission. In the present study, we analyzed using RNA-Seq, the lncRNAs that were differentially expressed in response to Rice black-streaked dwarf virus (RBSDV) infection in *Laodelphax striatellus* (Fallén) midgut. A total of 13,927 lncRNAs were identified and over 69% were assigned to intergenic regions. Among them, 176 lncRNAs were differentially expressed and predicted to target 168 *trans*-regulatory genes. Ten differentially expressed lncRNAs were selected and their expression changes were validated by RT-qPCR. KEGG analysis showed that these target genes were enriched in the essential biological process, such as Purine metabolism, Valine, leucine and isoleucine degradation, and Fatty acid elongation. The expression levels of the differentially expressed lncRNAs and the predicted target genes that were significantly enriched in the Human papillomavirus infection pathway were analyzed by RT-qPCR. The results showed that several lncRNAs were co-expressed with their target genes. One of the lncRNAs called MSTRG15394 and its target gene, encoding a secreted protease inhibitor (PI), were up-regulated at the transcriptional level after RBSDV infection. Knockdown of MSTRG15394 could down-regulate the PI expression at mRNA level. Inhibition of either MSTRG15394 or PI expression by RNA interference promoted RBSDV accumulation in *L. striatellus* midgut. Our finding provides new insights into the function of lncRNAs in regulating virus infection in an important insect vector.

## Introduction

Plant viruses can be transmitted by non-vector, such as grafting, and friction. It can also be transmitted by biotic vectors, such as insects, fungi and nematodes. Over 76% of described plant viruses are transmitted by insects, including whiteflies, aphids, planthoppers, leafhoppers, and thrips (Hogenhout et al., 2008; Jia et al., 2018). The manners of plant virus transmission via insect vectors can be divided into non-persistent, semi-persistent, or persistent manner (Ng and Falk, 2006). According to whether the virus replicates in their insect vector, the persistent viruses are transmitted in persistent circulative or persistent propagative manners (Jia et al., 2018). Persistent plant viruses move from esophagus to gut lumen, then to hemolymph or other tissues, and finally into salivary glands. The viruses are introduced back into the plant when the insects are feeding (Hogenhout et al., 2008). The insect midgut is a significant barrier for persistent viruses to enter the vector’s cells (Qin et al., 2018; Xia et al., 2018). Therefore, the investigation of the interaction between plant viruses and insect vectors in midgut is beneficial for understanding the mechanism of virus transmission.

The small brown planthopper, *Laodelphax striatellus* (Fallén), is a notorious rice insect. It causes a severe reduction in rice yield through direct sucking and the viral diseases that it transmits. One of the important viruses transmitted by *L. striatellus* is Rice black-streaked dwarf virus (RBSDV), which belongs to the genus *Fijivirus* of the family *Reoviridae* and is transmitted in a persistent propagative manner (Bai et al., 2001; Zhang et al., 2001; Wu et al., 2020). RBSDV only infects plants in the family Poaceae, such as *Oryza sativa*, *Triticum aestivum*, and *Zea mays*. The virus infected plants become severely stunted with darkened leaves and cannot head sprouting when infected at seedling stage (Zhang et al., 2001; Wang et al., 2009). RBSDV genome contains 10 segments (*S1*–*S10*) of double-stranded RNA (dsRNA) and encodes 13 proteins (Zhang et al., 2001; Wang et al., 2003; He et al., 2020). Each of the segments 5, 7, and 9 encodes two proteins, respectively. RBSDV P10 protein is the outer capsid protein, which plays crucial roles in the viral transmission process and can trigger endoplasmic reticulum stress in the plant (Liu et al., 2007; Sun Z. et al., 2013). P5-1, P6, and P9-1 proteins have been identified as the components of viral viroplasm, where the virus replicates and assembles (Sun et al., 2013b; He et al., 2020). Midgut is the first barrier to limit RBSDV transmission in *L. striatellus*. RBSDV needs to overcome midgut barriers, then move to hemolymph and finally spread to the salivary gland (Jia et al., 2014).

Long non-coding RNAs (lncRNAs) are defined as non-coding RNA transcripts that do not encode proteins. They can promote or suppress gene expression at transcriptional or post-translational levels by interacting with RNA, DNA or proteins (Mercer et al., 2009; Wu et al., 2012). LncRNAs are longer than 200 nucleotides, and transcribed by RNA polymerase II and polymerase III (Wu et al., 2012). Their expression levels are lower than protein encoding transcripts, and are not conserved between species (Necsulea et al., 2014). Previous studies have shown that lncRNAs participate in regulating the host innate immune system during virus infection. For example, lncRNA-155 targeted the protein tyrosine phosphatase 1B to modulate innate immunity against influenza A virus (IAV) infection in mouse (Maarouf et al., 2019). LncRNA-1317 was involved in the host anti-viral defense during dengue virus serotype 2 (DENV-2) infection in *Aedes aegypti* (Kayvan et al., 2016). In addition, lncRNA-SAF could regulate human immunodeficiency virus-1 (HIV-1) infection through activating apoptotic effector caspases in human macrophages (Boliar et al., 2019). However, few studies focus on the lncRNA expression profiles and their functions in the process of plant virus transmission via its insect vectors.

In the present research, the midgut of *L. striatellus*, which was a barrier for RBSDV infection, were dissected and used for RNA-Seq. We revealed the expression profiles of lncRNAs in RBSDV free and infected midgut samples and identified the differentially expressed lncRNAs. The expression of differentially expressed lncRNAs and their predicted target genes in Human Papillomavirus infection pathway were validated by RT-qPCR. In addition, the function of lncRNA MSTRG15394 in RBSDV infection was examined by double-stranded RNA (dsRNA) injection. These results provide a comprehensive foundation for understanding the roles of the lncRNAs in the regulation of virus infection in insect vectors.

## Materials and Methods

### Insect and Virus

The virus-free population of *L. striatellus* was collected from Haian (Jiangsu, China; 32.57°N, 120.45°E), and maintained in an incubator at 26 ± 1°C, humidity 70–80% and 16 h light: 8 h dark photoperiod. RBSDV infected rice plants with typical stunting symptoms were collected in the field.

The 3rd-instar *L. striatellus* nymphs were reared on RBSDV-infected rice plants for 2 days. Then, the nymphs were transferred to healthy rice seedlings. After 2 days later, the nymphs were collected as RBSDV-infected sample. Meanwhile, the 3rd-instar nymphs were reared on healthy rice seedlings for 4 days, and the nymphs were collected as virus-free sample.

### Midgut Collection and RNA Extraction

For midgut dissection, the collected nymphs were cooled on ice and then rinsed with 75% ethanol. After washing three times with sterilized-deionized water, the midgut was dissected with sterile forceps under stereomicroscope in chilled 1 × phosphate buffer solution (1 × PBS, pH 7.4). Midguts dissected from 200 virus-free or RBSDV-infected nymphs separately were used as virus-free and RBSDV-infected samples for RNA-Seq, and each sample had three independent biological replications.

Total RNA of the midgut was extracted using TRIzol (Invitrogen, United States) following the manufacturer’s instructions. The RNA quantity was measured by spectrophotometery (NanoDrop 2000, Thermo Fisher Scientific) and the quality was analyzed by agarose gel electrophoresis.

### RNA-Seq Sequencing and Bioinformatics Analysis

RNA-Seq library construction, Illumina high-throughput sequencing and sequencing reads assembly were performed by Lianchuan Biotechnology (Hangzhou, China). The sequencing data had been deposited in the Gene Expression Omnibus (GEO) at the National Center for Biotechnology Information (NCBI) under accession number GSE153102. The genome version of *L. striatellus* for transcripts mapping was ASM333518v2. The Cutadapt software was used to clean the raw sequencing reads, and the quality of the sequencing reads was evaluated and verified using software of FastQC^1^. Then, the Bowtie2 and Tophat2 were used to map reads to the assembled genome of *L. striatellus* (Langmead and Salzberg, 2012; Kim et al., 2013). The mapped reads of each sample were assembled using StringTie (Pertea et al., 2015). In order to identify lncRNAs, the transcripts which overlapped with known mRNAs and shorter than 200 bp were discarded and the coding potential of transcripts was predicted using CPC, CNCI and Pfam (Kong et al., 2007; Sun et al., 2013a; Bateman et al., 2004). All transcripts with CPC score < -1 and CNCI score < 0 were removed and the remaining transcripts with class code (i, j, o, u, x) were considered as lncRNAs. The class codes i, j, o, u, and x represented the transfrag falling entirely in intron, novel isoform containing at least one splice junction with reference transcript, exonic overlap with reference transcript, intergenic transcript, and exonic overlap with the opposite strand of reference transcript, respectively. StringTie software was used to analyze the expression level of mRNAs and lncRNAs through calculating the fragments per kilobase of exon model per million mapped fragments (FPKM) values (Pertea et al., 2015). The different expression analysis of mRNAs and lncRNAs was selected with log2 ^(foldchange)^ > 1 or log2 ^(foldchange)^ < −1 and with statistical significance (*p* < 0.05) by R package Ballgown (Frazee et al., 2015). The functions of differentially expressed lncRNAs were explored ground on the functional annotation of their related protein-coding genes through *cis*- and *trans*- analysis with perl script (Zhu et al., 2017; Ran et al., 2018). In *cis*, we performed co-expression analysis of the protein-coding genes 10 kb upstream and downstream of the differently expressed lncRNAs located at the same chromosome. lncRNAs could also be as *trans* regulators which regulated their target genes located at the different chromosome. We performed the expression levels of lncRNAs and protein-coding genes to investigate their co-expression relationships using Pearson correlations (FDR < 0.01; *p* < 0.01). Kyoto Encyclopedia of Genes and Genomes database (KEGG)^2^ pathway analysis was performed to provide insights into the reaction network and molecular interaction of the predicted differentially expressed lncRNA target genes.

### Quantitative Real-Time PCR (RT-qPCR)

Quantitative PCR reactions were performed using SYBR PrimeScript™ RT-PCR Kit (Takara, Japan) and then conducted on IQ™5 multicolor real-time PCR detection system (BIO-RAD, United States). One micrograms of total RNA were reverse transcribed to obtain cDNA using PrimeScript™ RT reagent kit with gDNA Eraser (Takara, Japan). Each RT-qPCR experiment performed with three independent biological replications, and each biological replication contained three independent technical replications. *LsRPL5* (encoding ribosome protein L5) was used as an internal reference control and data analysis was calculated through 2^–ΔΔCt^ method (Wu et al., 2019). The RT-qPCR primers were designed by Beacon Designer 7.7 software and listed in Supplementary Table S1.

### RNA Interference (RNAi)

Double-stranded RNA (dsRNA) was synthesized through T7 high yield transcription kit (Invitrogen, United States) according to the manufacturer’s instructions. The primers for dsRNA synthesis were listed in Supplementary Table S1. After anesthetized with carbon dioxide, the virus-free nymphs of 3rd-instar *L. striatellus* were immobilized on the 1% agarose plate. Approximately 100 nL dsRNA (2 μg/μL) was injected into the conjunction between prothorax and mesothorax of each nymph by FemtoJet microinjector (Eppendorf, Germany).

Thirty 3rd-instar nymphs were used for dsRNA injection of each gene, and three independent biological replications were carried out. The dsRNA of enhanced green fluorescent protein (EGFP) was injected as control. The dsRNA injected 3rd-instar nymphs were transferred to healthy rice seedlings. After one or 4 days, the midguts of nymphs were dissected and the interference efficiency was measured by RT-qPCR. To analyze the effect of lncRNA and its target gene on RBSDV accumulation, the 3rd-instar nymphs injected with dsRNA were fed on RBSDV-infected rice plants for 4 days, and then the midguts were collected. The expression changes of RBSDV *S10* and RBSDV replication related genes *S5-1*, *S6*, and *S9-1* were analyzed by RT-qPCR after dsRNA injection.

### Detection of RBSDV by Immunostaining

*L. striatellus* nymphs injected with dsRNA were transferred to RBSDV-infected rice plants for 4 days to acquire virus. The midguts of nymphs were dissected for immunofluorescence analysis. The midguts were washed with 1 × PBS and then fixed for 1 h using 4% paraformaldehyde. After washing with 1 × PBS, the midguts were permeabilized with 2% Triton X-100 for 30 min and blocked with 3% BSA for 2 h. Then, the midguts were incubated with primary antibody (anti-RBSDV P10 mouse mAb conjugated with FITC) overnight at 4°C, and incubated with TRITC phalloidin for 10 min (Solarbio, China). Finally, the midguts were viewed with LSM 710 (ZEISS, Germany) for confocal imaging.

### Statistical Analysis

Statistical analysis was performed using SPSS 20.0 (IBM Corporation, United States). The relative expression levels of genes were analyzed using one-way analysis of variance (ANOVA) with least significant difference (LSD) test. The *p* < 0.05 and *p* < 0.01 were regarded as the threshold for significant and very significant difference, respectively.

## Results

### Expression Profiles of LncRNAs and mRNAs in Midgut of *L. striatellus*

RNA-Seq sequencing was performed to identify lncRNAs and mRNAs after RBSDV infection in *L. striatellus* midgut. Over 20 and 21 million clean reads from virus-free (VF) and RBSDV infected (RB) libraries were obtained, respectively. Q30 base percentages of each sequencing sample were no <93.89%, indicating the quality of the sequencing was sufficient. More than 50.37% of the clean reads were perfectly mapped to the *L. striatellus* genome. The uniquely mapping ratio was ranged from 48.03 to 52.08% (Table 1).

**TABLE 1:** Summary of reads mapping to the reference genome.

Samples	Raw reads	Clean reads	Q30	Mapped reads	Mapping ratio	Uniquely mapped reads	Uniquely mapping ratio
VF^&^ 1	93,825,650	68,473,008	94.10%	37,511,142	54.78%	35,661,647	52.08%
VF^&^ 2	106,770,512	74,096,390	94.62%	37,320,379	50.37%	35,588,105	48.03%
VF^&^ 3	85,605,160	65,086,282	95.18%	35,644,267	54.76%	33,782,316	51.90%
RB^#^ 1	98,956,596	83,649,858	93.89%	42,361,233	50.64%	40,311,612	48.19%
RB^#^ 2	100,209,506	71,263,346	94.77%	36,664,950	51.45%	35,049,085	49.18%
RB^#^ 3	89,898,002	62,707,912	95.11%	33,533,899	53.48%	31,857,015	50.80%

**^&^VF, virus-free; ^#^RB, RBSDV infected.**

After a stringent filtering process, a total of 13,927 novel lncRNAs were identified from VF and RB libraries (Supplementary Table S2). According to the cuffcompare classes, most of the lncRNAs (69.45–70.10%) aligned to intergenic regions (u) (Supplementary Figure S1). In addition, 35,316 mRNAs were assembled after clustering and filtering out low-quality sequences (Supplementary Table S3).

**FIGURE 1:**
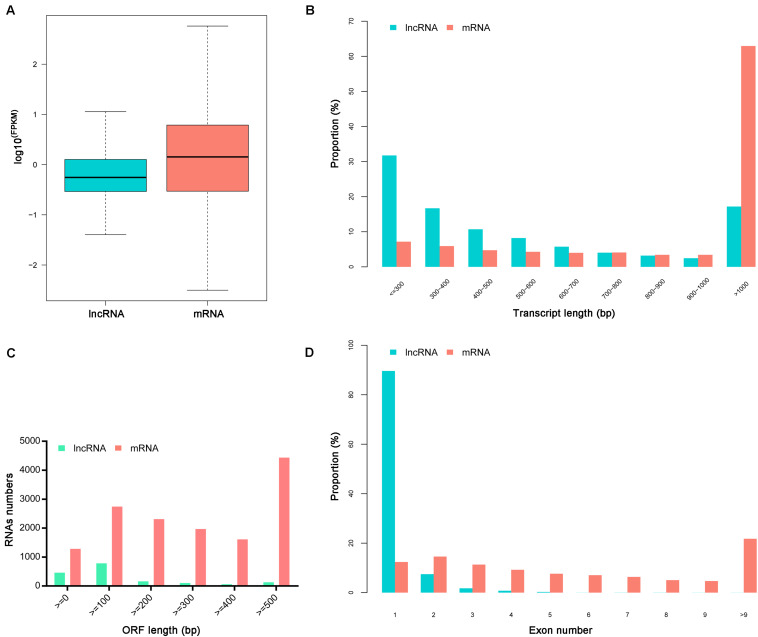
Characteristics of identified lncRNAs and mRNAs. **(A)** The expression levels of mRNAs and lncRNAs transcripts indicated by log10 ^(FPKM)^. **(B)** The length distributions of lncRNAs and mRNAs transcripts. **(C)** The open reading frame (ORF) length of lncRNAs and mRNAs**. (D)** The number of exons per transcript for lncRNAs and mRNAs.

The ratio of lncRNA vs. mRNA amounts showed that the average expression levels of lncRNAs were lower than those of mRNAs (Figure 1A). The mean length of lncRNAs was 734 bp and 17.19% of lncRNAs were over 1,000 bp. The lncRNAs transcripts were shorter than protein-coding mRNAs (Figures 1B,C). Besides, the identified lncRNAs had fewer exons than mRNAs, and approximately 89.62% of the lncRNAs had only one exon (Figure 1D).

### Differentially Expressed LncRNAs and mRNAs

**FIGURE 2:**
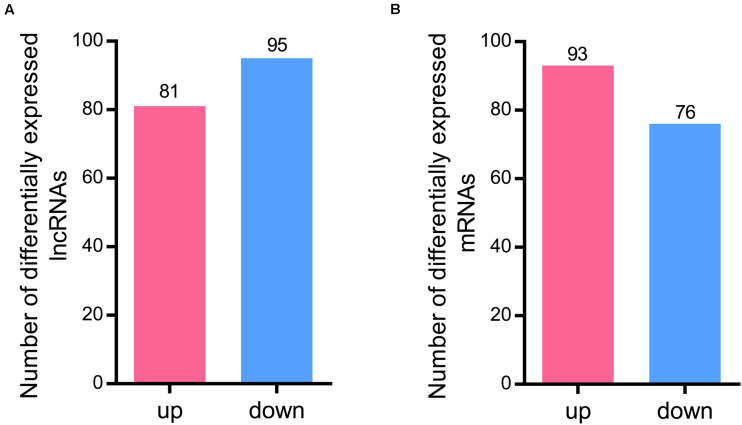
Differentially expressed lncRNAs and mRNAs identified in *L. striatellus* midgut after RBSDV infection. **(A)** The number of up and down differentially expressed lncRNAs. **(B)** The number of identified differentially expressed mRNAs.

A total of 176 differentially expressed lncRNAs were identified after RBSDV infection in *L. striatellus* midgut. Among them, 81 lncRNAs were up-regulated while 95 were down-regulated in RBSDV infected midgut (Supplementary Table S4 and Figure 2A). Meanwhile, 169 differentially expressed mRNA transcripts were also identified after RBSDV infection, including 93 up-regulated and 76 down-regulated transcripts (Supplementary Table S5 and Figure 2B).

**FIGURE 3:**
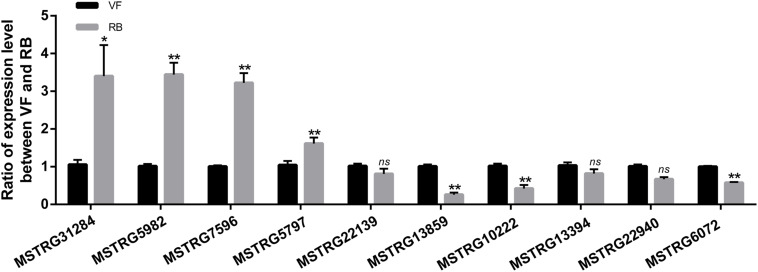
Validation of the expression changes of 10 differentially expressed lncRNAs through RT-qPCR after RBSDV infection. The expressions of 10 differentially expressed lncRNAs were analyzed through 2^− ΔΔCt^ method after RBSDV infection. The *ns* represents no significant difference, and asterisks (*) and (**) indicate significant differences at *p* < 0.05 and *p* < 0.01 levels, respectively.

To validate the sequencing data, the expression changes of five up-regulated and five down-regulated lncRNAs were analyzed after RBSDV infection in *L. striatellus* midgut using RT-qPCR. As shown in Figure 3, the expression changes of seven transcripts were consistent with transcriptome data. However, the expression levels of MSTRG22139, MSTRG13394 and MSTRG22940 had no significant differences after virus infection.

### Prediction of Target Genes for Differentially Expressed LncRNAs

LncRNAs have been reported to regulate gene expression via *cis*- or *trans*-acting regulation (Zhan et al., 2016; Ran et al., 2018). To analyze the functions of lncRNAs, the *cis*- and *trans*-acting target genes of the differentially expressed lncRNAs were identified. In total, 176 differentially expressed lncRNAs were predicted to *trans*-regulate 168 differentially expressed mRNA transcripts (Supplementary Table S6). No *cis*-acting target genes were predicted to be regulated by these differentially expressed lncRNAs.

**FIGURE 4:**
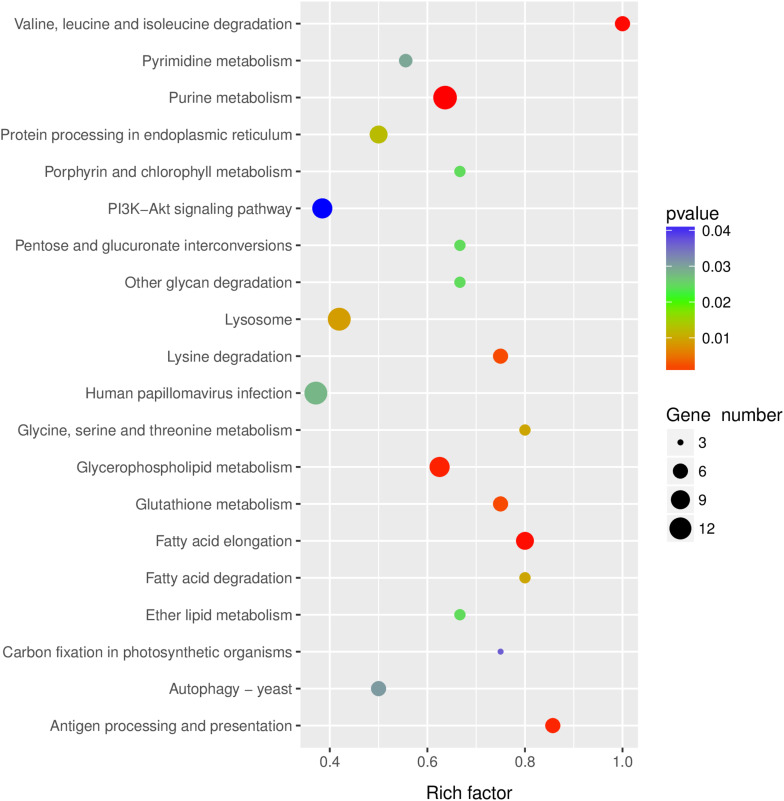
KEGG pathway analysis of the predicted lncRNAs target genes. Top 20 enriched pathways for the predicted lncRNAs target genes were analyzed.

KEGG pathway analysis was performed to gain insights into the function of differentially expressed lncRNA target genes. The top twenty significantly enriched pathways of the *trans*-regulated target genes were presented in Figure 4. Purine metabolism, Valine, leucine and isoleucine degradation, Fatty acid elongation, Glycerophospholipid metabolism and Antigen processing and presentation were the five most significantly enriched pathways (Figure 4).

### Human Papillomavirus Infection Pathway in Response to RBSDV Infection

**FIGURE 5:**
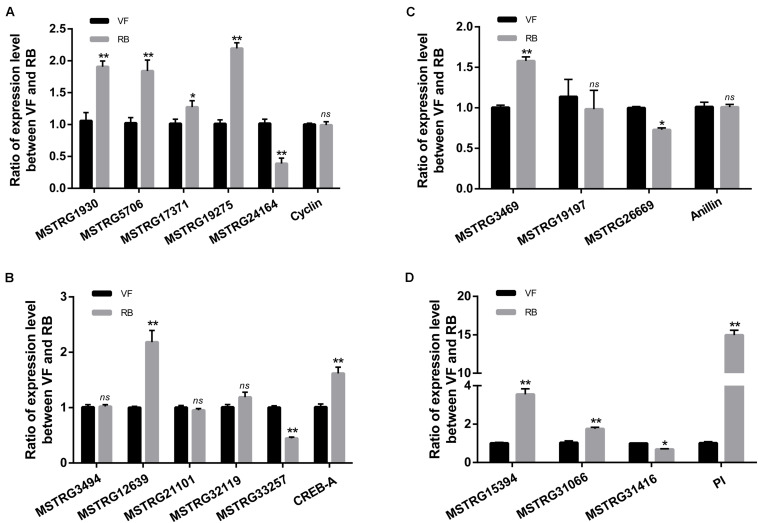
Validation of the expression of 16 differentially expressed lncRNAs and four predicted target genes in Human papillomavirus infection pathway by RT-qPCR. **(A–D)** The relative expression levels of four target genes and their lncRNAs, cyclin **(A)**, CREB-A **(B)**, anillin **(C)**, and PI **(D)**. The *ns* represents no significant difference, and asterisks (*) and (**) indicate significant differences at *p* < 0.05 and *p* < 0.01 levels, respectively.

The pathway of Human papillomavirus infection, which related to virus infection, was also significantly enriched after RBSDV infection by KEGG pathway analysis (Figure 4). A total of sixteen differentially expressed lncRNAs and their four co-expressed target genes were identified in this pathway. Fourteen lncRNAs were up-regulated and two lncRNAs were down-regulated after RBSDV infection. The four target genes identified as part of this pathway were all up-regulated (Table 2). The sixteen lncRNAs were divided to four groups according to their co-expressed target genes. The expression changes of sixteen lncRNAs and their corresponding co-expressed target genes were verified by RT-qPCR. Results showed that the expression changes of eight lncRNAs and two target genes were consistent with the sequencing data (Figure 5). The expression levels of two lncRNAs (MSTRG12639 and MSTRG33257) and their target gene cyclic AMP response element-binding protein A (CREB-A) were significantly changed after RBSDV infection (Figure 5B). The expression levels of three lncRNAs (MSTRG15394, MSTRG31066 and MSTRG31416) and their target gene protease inhibitor (PI) were also significantly changed after RBSDV infection (Figure 5D). However, the expression levels of target genes cyclin and anillin, which were the predicted target genes for the other eight lncRNAs, had no significant changes after RBSDV infection (Figures 5A,C). These results indicated that the Human papillomavirus infection pathway might be involved in RBSDV infection of *L. striatellus* midgut.

**TABLE 2:** Summary of differentially expressed lncRNAs and its predicted target genes involving in Human papillomavirus infection pathway.

LncRNA id	Length	log2(fc)	*P*-value	Regulation	Target description
MSTRG1930	812	1.93	0.02	Up	Cyclin
MSTRG5706	992	1.05	0.02	Up	Cyclin
MSTRG17371	2500	1.19	0.04	Up	Cyclin
MSTRG19275	509	1.32	0.05	Up	Cyclin
MSTRG24164	680	1.08	0.02	Up	Cyclin
MSTRG3494	523	1.17	0.01	Up	CREB-A
MSTRG12639	4043	3.08	0.01	Up	CREB-A
MSTRG21101	633	-1.19	0.05	Down	CREB-A
MSTRG32119	1204	1.42	0.01	Up	CREB-A
MSTRG33257	833	1.41	0.02	Up	CREB-A
MSTRG3469	997	1.55	0.03	Up	Anillin
MSTRG19197	548	-1.42	0.00	Down	Anillin
MSTRG26669	1083	2.08	0.02	Up	Anillin
MSTRG15394	653	1.28	0.02	Up	Secreted protease inhibitor
MSTRG31066	787	1.08	0.01	Up	Secreted protease inhibitor
MSTRG31416	913	1.17	0.05	Up	Secreted protease inhibitor

### MSTRG15394 and Secreted Protease Inhibitor Co-expression Analysis

**FIGURE 6:**
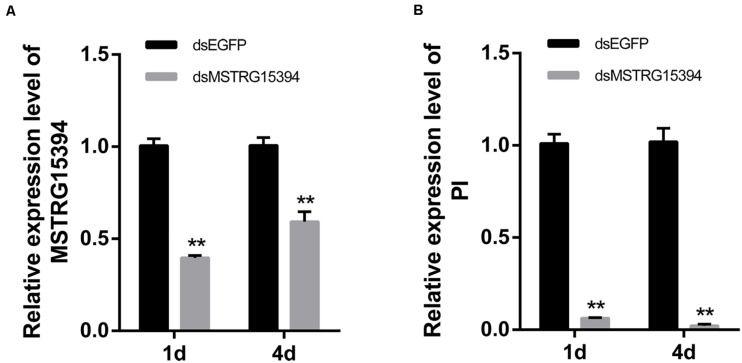
Co-expression analysis of MSTRG15394 and PI after knockdown of MSTRG15394 by RT-qPCR. **(A)** The expression of MSTRG15394 was significantly reduced on one and 4 days after RNAi treatment. **(B)** The expression of PI was significantly decreased on one and 4 days after knockdown of MSTRG15394. The asterisks (**) indicate significant differences at *p* < 0.01 level.

Both MSTRG15394 and PI were significantly expressed after RBSDV infection. PI, in particular, increased about 15-fold after RBSDV infection. To confirm whether MSTRG15394 regulates the expression of PI, we knocked down the expression of MSTRG15394 by RNA interference (RNAi) and analyzed its effect on the expression level of PI. The expression of MSTRG15394 reduced to 40.77 and 60.33% after knockdown of MSTRG15394 for one and 4 days, respectively (Figure 6A). Knockdown of MSTRG15394 reduced the expression of PI to 93.67 and 97.78% on one and four days after RNAi, respectively (Figure 6B). The results indicated that MSTRG15394 could regulate the expression of PI at the mRNA level.

### MSTRG15394 and Secreted Protease Inhibitor Regulated RBSDV Infection in *L. striatellus* Midgut

**FIGURE 7:**
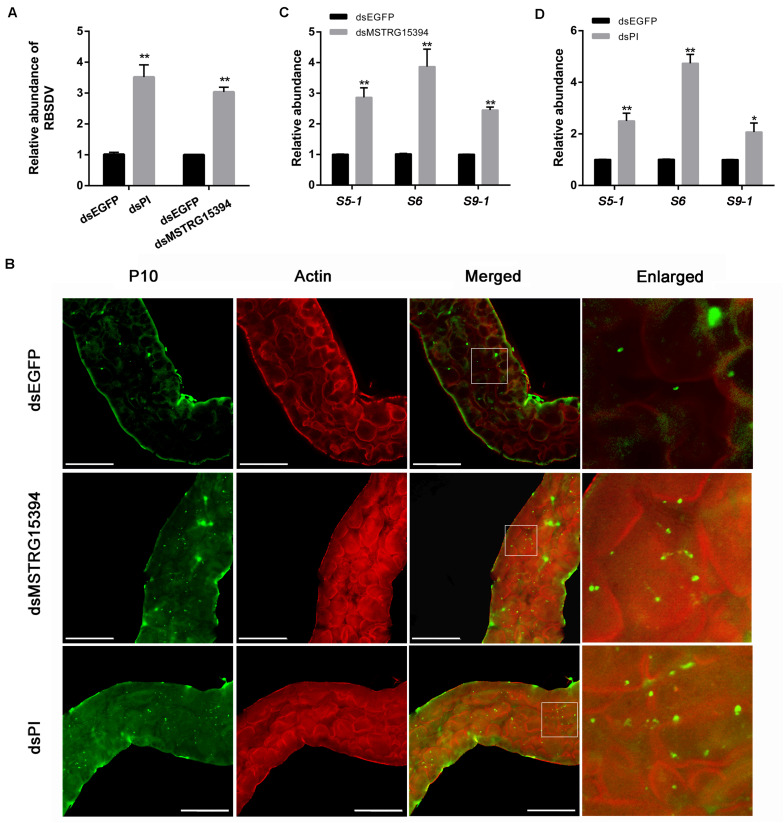
Knockdown of MSTRG15394 or PI increased RBSDV accumulation in *L. striatellus* midgut. **(A)** RT-qPCR was used to analyze the expression of RBSDV *S10* gene. The results showed that the relative abundance of RBSDV was significantly increased in *L. striatellus* midgut after knockdown of MSTRG15394 or PI. **(B)** Immunofluorescence analysis showed that the relative abundance of RBSDV labeled as green dots was significantly increased when MSTRG15394 or PI was knocked down. **(C,D)** The relative expression levels of *S5-1*, *S6*, and *S9-1* genes were significantly increased after knockdown of MSTRG15394 **(C)** or PI **(D)**. The asterisks (*) and (**) indicate significant differences at *p* < 0.05 and *p* < 0.01 levels, respectively. Scale bars, 50 μm.

In order to analysis the roles of MSTRG15394 and PI during RBSDV infection of *L. striatellus* midgut, we knocked down MSTRG15394 and PI by injecting dsRNA into *L. striatellus* via microinjection. The expression level of PI was significantly decreased on 1 and 4 days after dsRNA injection, respectively (Supplementary Figure S2). Furthermore, knockdown of either MSTRG15394 or PI resulted in a significant increase of RBSDV *S10* gene by RT-qPCR in *L. striatellus* midgut (Figure 7A). Similar results were obtained when the virus accumulation in *L. striatellus* midgut was detected by immunofluorescence after knockdown of MSTRG15394 or PI (Figure 7B). Besides, we found that the expression levels of RBSDV replication related genes, *S5-1*, *S6*, and *S9-1*, were significantly up-regulated after knockdown of MSTRG15394 or PI (Figures 7C,D). In order to eliminate the effect of dsRNA injection on the ability of *L. striatellus* to acquire virus from RBSDV infected rice plants, the viruliferous 3rd-instar nymphs which reared on RBSDV-infected rice plants for 2 days were used to inject dsRNA of MSTRG15394 and PI. Then, the nymphs were transferred to healthy rice plants for 3 days. The RBSDV abundance was also significantly increased after knockdown of either MSTRG15394 or PI by RT-qPCR (Supplementary Figure S3), indicating dsRNA injection had no effect on the ability of *L. striatellus* to acquire RBSDV. In conclusion, these data suggested that MSTRG15394 and PI could inhibit the accumulation and replication of RBSDV in *L. striatellus* midgut.

## Discussion

LncRNAs play vital roles in biological processes in mammals (Mercer et al., 2009). It is reported that lncRNAs are involved in the regulation of viral infection in human. For example, the relative expression of lncRNA-CMPK2 was up-regulated in human liver during *hepatitis C virus* (HCV) infection and involved in negative regulation of the interferon (IFN) response (Kambara et al., 2014). However, the functional studies of lncRNAs in insects are still at preliminary stages. In recent years, numerous lncRNAs have been identified in insect by high-throughput sequencing, especially in *Drosophila melanogaster*, *Anopheles gambiae*, and *Apis mellifera* (Young et al., 2012; Humann et al., 2013; Jenkins et al., 2015). A part of lncRNAs have been identified in *L. striatellus* after *Rice stripe virus* (RSV) infection, but the profiles and functions of lncRNAs are still unknown, especially in the midgut (Chen et al., 2019).

The midgut is a barrier for persistent circulative plant viruses to invade the insect vector (Nagata et al., 2002; Hurd et al., 2003; Qin et al., 2018; Xia et al., 2018). The analysis of lncRNAs in *L. striatellus* midgut is helpful to obtain specific lncRNAs related to virus infection. In the current study, a total of 13,927 lncRNAs existing in *L. striatellus* midgut, were systematically identified and characterized. Comparing with protein coding transcripts, the lncRNAs revealed in this study had lower expression levels, shorter lengths and fewer exons. These results were similar to the previous lncRNA research on insect species (Wu et al., 2016; Zhu et al., 2017; Wang et al., 2018). All the lncRNAs identified in VF or RBSDV infected midgut are novel, suggesting lncRNAs are specific in *L. striatellus* midgut or other tissues. Another reason for not identifying known lncRNAs is that few lncRNAs had been previously identified in *L. striatellus*. Among 81 up-regulated and 95 down-regulated lncRNAs, we validated the expression changes of 10 lncRNAs, and the results indicated that the accuracy of RNA-Seq analysis was robust and was consistent with previous research (Zhu et al., 2017).

LncRNAs regulate gene expression and protein synthesis in many different ways, such as chromatin modification, transcription and post-transcriptional processing (Zhu et al., 2017; Ran et al., 2018). Identification of LncRNA target genes and exploring their regulatory effects is critical for elucidating the function of lncRNAs. To reveal the possible function of lncRNAs, *cis*- and *trans*-target genes and differentially expressed mRNAs were subjected to bioinformatics analyses. In contrast to the target genes identified from some model insects (Mercer et al., 2009; Young et al., 2012), only *trans*-target genes were predicted from the differentially expressed lncRNAs in our study. This may because only 1.26% of lncRNAs were differentially expressed after RBSDV infection in *L. striatellus* midgut. We speculated that only a part of lncRNAs played vital roles in RBSDV infection through *trans*-regulated manner. A majority of lncRNAs, not differentially expressed in RBSDV infection, might play an important role in other biological processes in *L. striatellus* via *cis*- or *trans*-regulated manners. A total of 168 *trans*-target genes were detected. Similar with the previous studies on KEGG pathway analysis (Liu et al., 2018; Ran et al., 2018), target genes were enriched in multiple basic biological processes, such as Glycerophospholipid metabolism, Purine metabolism and so on.

Human papillomavirus infection pathway was also significantly enriched during RBSDV infection in *L. striatellus* midgut. This pathway is pivotal in host immune response to virus infection, and is closely related to other pathways, such as Wnt signaling pathway, Toll-like receptor signaling pathway, mTOR signaling pathway and JAK-STAT signaling pathway (Zhou et al., 2011). Therefore, Human papillomavirus infection pathway might be played a crucial role in RBSDV infection. Sixteen differentially expressed lncRNAs and their four predicted target genes were identified in this pathway. The expression of these differentially expressed lncRNAs and their target genes were analyzed after RBSDV infection. The results indicated that MSTRG12639 and MSTRG33257 might be co-expressed with CREB-A, while MSTRG15394, MSTRG31066 and MSTRG31416 might be co-expressed with PI. CREB is a well-known transcription factor which responds rapidly to stress signals, growth factors, neurotransmitters and so on (Shaywitz and Greenberg, 1999; Mayr and Montminy, 2001). It can activate the transcription of over 5,000 target genes and plays essential roles in cellular growth, lipid metabolism and gluconeogenesis (Ahn et al., 1998; Servillo et al., 2002). CREB-mediated transcription can be activated by *Hepatitis B virus* (HBV) through interacting with CREB-binding protein (Cougot et al., 2007). PI plays vital roles in antivirus and preventing carcinogenesis (Kennedy, 1998; Bruno et al., 2006). The results suggested that the differentially expressed lncRNAs in Human papillomavirus infection pathway might be involved in the RBSDV infection in *L. striatellus* midgut through regulating their target genes.

The function of MSTRG15394 and its target gene PI were further studied using RNAi method. Inhibition of the expression of MSTRG15394 led to a significant decrease in the expression of PI, suggesting that MSTRG15394 could regulate the expression of PI. On the one hand, MSTRG15394 may be acting as a competing endogenous RNA (ceRNA) and exerted the roles of post-transcriptionally regulation of PI through absorbing miRNAs which combined with PI. On the other hand, MSTRG15394 might be regulated by the transcriptional activation of PI by recruiting transcriptional/splicing factors. Furthermore, knockdown of MSTRG15394 and PI caused an increase of RBSDV accumulation in *L. striatellus* midgut. The results indicate that MSTRG15394 and PI have antivirus functions and this is the first report that lncRNA regulates the RBSDV infection in *L. striatellus*.

In summary, we identified the differentially expressed lncRNAs in *L. striatellus* midgut after RBSDV infection. The effects of MSTRG15394 and PI on virus accumulation were analyzed by RNAi and immunostaining. The results indicate that lncRNAs play key roles in the process of RBSDV infection in an insect vector.

## Conclusion

In conclusion, this research reveals the differentially expressed lncRNAs that are responding to a dsRNA virus (RBSDV) infection in the *L. striatellus* midgut. The predicted target genes of these differentially expressed lncRNAs are enriched in the essential biological process, such as Purine metabolism, Valine, leucine and isoleucine degradation, and Fatty acid elongation. Furthermore, knockdown of lncRNA MSTRG15394 down-regulates the PI expression at mRNA level. Inhibition of MSTRG15394 expression or its target gene PI promotes the RBSDV accumulation in *L. striatellus* midgut. Our data contribute to understanding the function of lncRNA in the regulation of viral infection.

## Data Availability Statement

The sequencing data has been deposited into the GEO (accession: GSE153102, link: https://www.ncbi.nlm.nih.gov/geo/query/acc.cgi?acc=GSE153102).

## Author Contributions

J-HZ and Q-FX conceived the study. J-HZ, YD, WW, D-SY, MW and H-TW conducted the experiments. J-HZ and YD analyzed the data. All authors contributed to the article and approved the submitted version.

## Conflict of Interest

The authors declare that the research was conducted in the absence of any commercial or financial relationships that could be construed as a potential conflict of interest.

## Footnotes

1 http://www.bioinformatics.babraham.ac.uk/projects/fastqc/

2 http://www.kegg.jp

## Supplementary Material

FIGURE S1 The types of identified lncRNAs. The types of lncRNAs identified in three virus free (VF)samples **(A–C)**. The types of lncRNAs identified in three RBSDV infected (RB) samples **(D–F)**.

FIGURE S2 Expression analysis of PI after dsRNA treatment by RT-qPCR. The asterisks (^∗∗^) indicate significant differences at *p* < 0.01 level.

FIGURE S3 Knockdown of MSTRG15394 or PI increased RBSDV accumulation in *L. striatellus* midgut. The asterisks (^∗∗^) indicate significant differences at *p* < 0.01 level.

TABLE S1 Primers used in this study.

TABLE S2 The data of 13,927 novel lncRNAs identified from VF and RB libraries.

TABLE S3 The data of 35,316 mRNAs identified from VF and RB libraries.

TABLE S4 The data of 176 lncRNAs which were differentially expressed after RBSDV infection in *L. striatellus* midgut.

TABLE S5 The data of 169 mRNA transcripts which were differentially expressed after RBSDV infection in *L. striatellus* midgut.

TABLE S6 The data of *trans*-regulated target genes by differentially expressed lncRNAs after RBSDV infection in *L. striatellus* midgut.
