# Supplementary figures and images for: Comprehensive Identification and Characterization of Long Non-coding RNAs Associated With Rice Black-Streaked Dwarf Virus Infection in Laodelphax striatellus (Fallén) Midgut
Source: Front Physiol. 2020 Aug 12;11:1011. doi: 10.3389/fphys.2020.01011 (PMC7437459; doi:10.3389/fphys.2020.01011)

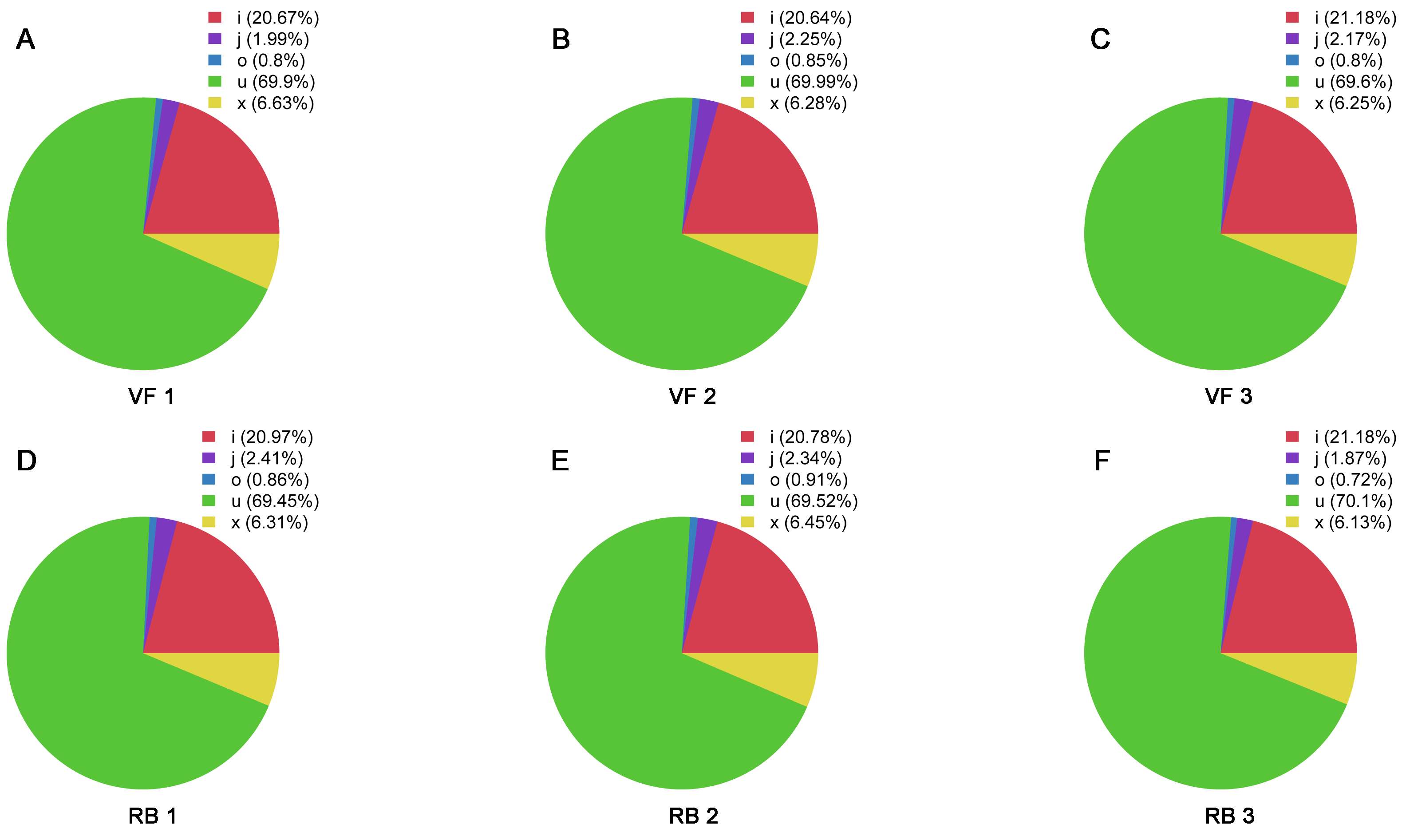

Supplement: FIGURE S1 — The types of identified lncRNAs. The types of lncRNAs identified in three virus free (VF)samples (A–C). The types of lncRNAs identified in three RBSDV infected (RB) samples (D–F). [file Image_1.TIF]

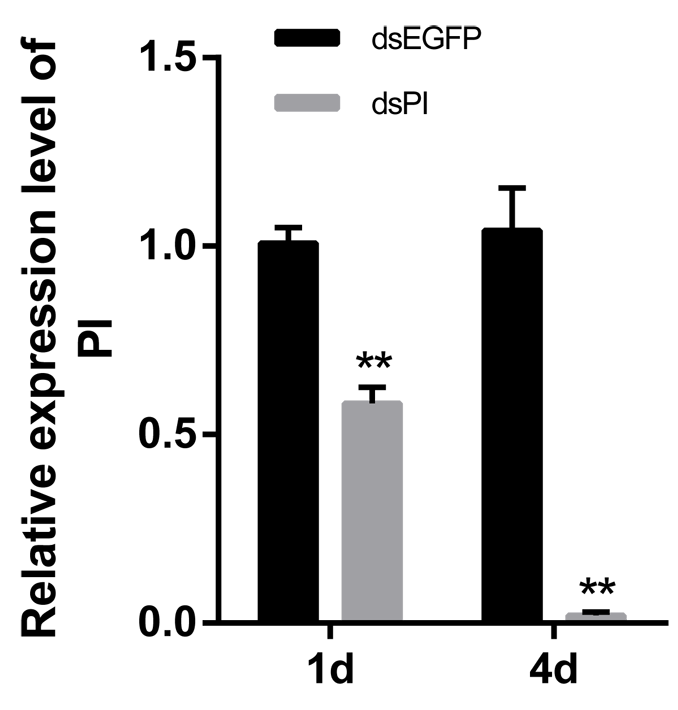

Supplement: FIGURE S2 — Expression analysis of PI after dsRNA treatment by RT-qPCR. The asterisks (∗∗) indicate significant differences at p < 0.01 level. [file Image_2.TIF]

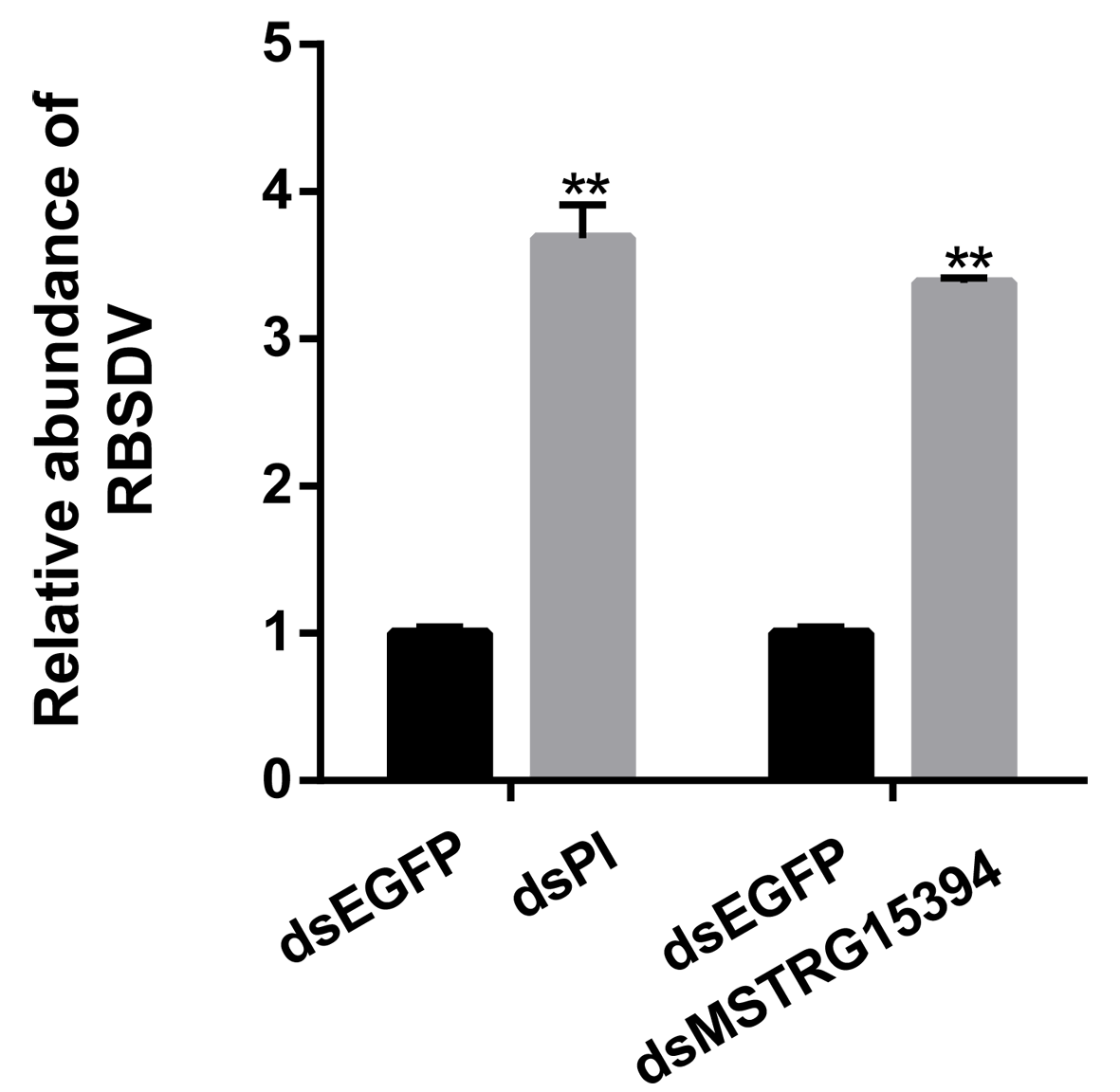

Supplement: FIGURE S3 — Knockdown of MSTRG15394 or PI increased RBSDV accumulation in L. striatellus midgut. The asterisks (∗∗) indicate significant differences at p < 0.01 level. [file Image_3.TIF]
