# Supplementary material for: Comprehensive Identification and Characterization of Long Non-coding RNAs Associated With Rice Black-Streaked Dwarf Virus Infection in Laodelphax striatellus (Fallén) Midgut
Source: Front Physiol. 2020 Aug 12;11:1011. doi: 10.3389/fphys.2020.01011 (PMC7437459; doi:10.3389/fphys.2020.01011)
Supplement: TABLE S1 — Primers used in this study. [file Table_1.DOCX]

**Table S1**. Primers used in this study.

| Gene name | Forward primer | Reverse primer |
| --- | --- | --- |
| MSTRG31284 | TTCATTCACAACAGCACATT | TCCAACCACACAGTAAAATAAG |
| MSTRG5982 | TCAGAGGCGATTGGTATTAC | GATGGTCCAACAGAGAAGAT |
| MSTRG7596 | CAGTGAATGACAGACGAATC | AACTAGAGAACCGATCCATC |
| MSTRG5797 | CATACTCACCTCACTGGATA | ATCTGTATTGGATGTCATTGTT |
| MSTRG22139 | AATTCGAGCATTCTAACACA | ATTCCATACAGCCAATACTC |
| MSTRG13859 | AATCGGTAGTTGCTGTATCT | AGTAAGGATGTTGGCTAGAG |
| MSTRG10222 | CGTTCTGGATGTCACCTATT | TTCGTCACTGGAATGATTGG |
| MSTRG13394 | CAAGAGACTTAGTGATGAAGAG | ATGGTATCAGGTAGAGTTATGT |
| MSTRG22940 | GACAACAACAAGTGAACATAC | AATCGGATAATAACGCATAGT |
| MSTRG6072 | AAACAACTGAAAGGATATGC | GATATGGAGAATGGAGGTTC |
| Cyclin | CAAGAGAACCAACAGCAATT | TCTCCTCGATTGTCCTTTAG |
| MSTRG1930 | CGTACTTGTTATATTGCGTAAG | AATTTGTTGCGAGGCTAATC |
| MSTRG5706 | CTTTCTGTGTCTGTTTCGTA | CACTCTGTATAATAAGTCCTGTT |
| MSTRG17371 | GTCCATAGTAACAGTGATAGAAC | TGCCAGAAGAACAAGTAGAA |
| MSTRG19275 | CTCACAAAGTTCTCCACAAC | CCATTCATAAGGCATCAACTG |
| MSTRG24164 | TTTGGCTGTTGGTTTATTGA | TTGAATTGTAGTATGAACTGGAT |
| CREB-A | TCTGTGGACAAATCGTTCTA | CATCCGTCACCATAAAGAGT |
| MSTRG3494 | TGATAGAAGAATGGCTGAGA | GGCGAACAAACCTAATAGAT |
| MSTRG12639 | CAACTTATGATGAGGCAATGA | TTTACTCTGGTCTCCACTTC |
| MSTRG21101 | TTTCTAACTAGGGACACTCAT | CACTAATCACTATCTGCTTACA |
| MSTRG32119 | GATTAACCACGGTCAACTTA | CACTTTCAACTTTGGCAATT |
| MSTRG33257 | AAATCTATTGTCCACCACTTT | TCTCTAACTCTAATCATTCTTGTG |
| Anillin | GTATTCCGAAGTTCTACTACAAG | AGACATTGGACGACTGATAT |
| MSTRG3469 | CTGGAATGTTGTGAGTCAATA | AATGGTATAACTAGCTGGTAAC |
| MSTRG19197 | CTTACGGTATAACATGCTCATC | GCCTGCTGAAATTGATAGAG |
| MSTRG26669 | AAATTGTTGAACTCGCTCTG | ATTCTCTTGAACTCCACTGT |
| PI | TTGCTCTCCTTATTGCTTCC | CTTGTCATCTTCGTGGTTGT |
| MSTRG15394 | CAGGATACAAGATATGAGAATAGC | ATTAAGTAAGACGGCAAAGC |
| MSTRG31066 | TGCGGATATTATCTTAATTCTCT | AATTCCATGTTGTAATCATTCG |
| MSTRG31416 | GTAGATGAGAAGTATGAGAGTGA | GCAAATAACTGAGGTTCCTT |
| S10  S5-1  S6  S9-1  RPL5  dsMSTRG15394 | GCCCCACGTTGCATCTTC  GTTTACGGTGGTGCAATTTTCA  GAACAGATTGCCAAATACGAAACA  TGGTGCTTCTCGTCAAACTGTCT  CCGAAGTGACAGGCGAGGAG  TAATACGACTCACTATAGGGTTGCTATCTCATCAAAGGCTA | TGTTGGGCAAAGTGCTAGTTTC  AGGCTTTCCTTCACTAACTTCTGACT  AAGAGCAGTACGCGTCGTTCA  GCCAACAATTCGTGTCCTGAA  CACGCTGTGCGGGATGTT  TAATACGACTCACTATAGGGGCGTTAAATTCTGGACGAAAA |
| dsPI | TAATACGACTCACTATAGGGATGCCTATTTCTATTTTCTCG | TAATACGACTCACTATAGGGTGTTCGCACTTGTCATCTTCG |
| dsEGFP | TAATACGACTCACTATAGGGAAGTTCAGCGTGTCCG | TAATACGACTCACTATAGGGCACCTTGATGCCGTTC |
